# Differential intracellular distribution of DNA complexed with polyethylenimine (PEI) and PEI-polyarginine PTD influences exogenous gene expression within live COS-7 cells

**DOI:** 10.1186/1479-0556-5-11

**Published:** 2007-11-26

**Authors:** Stephen R Doyle, Chee Kai Chan

**Affiliations:** 1 Department of Genetics and Human Variation, La Trobe University, Melbourne, Victoria 3086, Australia

## Abstract

**Background:**

Polyethylenimine (PEI) is one of the most efficient and versatile non-viral vectors available for gene delivery. Despite many advantages over viral vectors, PEI is still limited by lower transfection efficiency compared to its viral counterparts. Considerable investigation is devoted to the modification of PEI to incorporate virus-like properties to improve its efficacy, including the incorporation of the protein transduction domain (PTD) polyarginine (Arg); itself demonstrated to facilitate membrane translocation of molecular cargo. There is, however, limited understanding of the underlying mechanisms of gene delivery facilitated by both PEI and PEI-bioconjugates such as PEI-polyarginine (PEI-Arg) within live cells, which once elucidated will provide valuable insights into the development of more efficient non-viral gene delivery vectors.

**Methods:**

PEI and PEI-Arg were investigated for their ability to facilitate DNA internalization and gene expression within live COS-7 cells, in terms of the percentage of cells transfected and the relative amount of gene expression per cell. Intracellular trafficking of vectors was investigated using fluorescent microscopy during the first 5 h post transfection. Finally, nocodazole and aphidicolin were used to investigate the role of microtubules and mitosis, respectively, and their impact on PEI and PEI-Arg mediated gene delivery and expression.

**Results:**

PEI-Arg maintained a high cellular DNA uptake efficiency, and facilitated as much as 2-fold more DNA internalization compared to PEI alone. PEI, but not PEI-Arg, displayed microtubule-facilitated trafficking, and was found to accumulate within close proximity to the nucleus. Only PEI facilitated significant gene expression, whereas PEI-Arg conferred negligible expression. Finally, while not exclusively dependant, microtubule trafficking and, to a greater extent, mitotic events significantly contributed to PEI facilitated gene expression.

**Conclusion:**

PEI polyplexes are trafficked by an indirect association with microtubules, following endosomal entrapment. PEI facilitated expression is significantly influenced by a mitotic event, which is increased by microtubule organization center (MTOC)-associated localization of PEI polyplexes. PEI-Arg, although enhancing DNA internalization per cell, did not improve gene expression, highlighting the importance of microtubule trafficking for PEI vectors and the impact of the Arg peptide to intracellular trafficking. This study emphasizes the importance of a holistic approach to investigate the mechanisms of novel gene delivery vectors.

## Background

Gene therapy has the potential to treat many inherited and acquired genetic diseases. While applications of non-viral gene delivery are routinely used for a vast range of protocols within the general research environment, the progression to clinical therapeutic applications remains elusive. The realization of such a therapeutic approach is hampered by the lack of understanding of the mechanisms by which gene delivery vectors actually function. Despite similarities between vectors in terms of a typical gene delivery strategy, which are acknowledged to include: the packaging of exogenous DNA, specific targeting to cells and/or tissue, cellular uptake through the plasma membrane, intracellular transport, and finally nuclear import and transcription of the exogenous DNA into therapeutic products, all vectors have discrete characteristics that must be initially optimised for each application. Despite the widespread use of commercially available gene delivery vectors for basic science, researchers often are content to have a vector that simply works, and not question the fundamental delivery mechanisms of the vector itself.

The cationic polymer polyethylenimine (PEI) is among the most efficient and versatile non-viral vector (for reviews see [1,2]), and has been shown to be effective for DNA delivery both *in vitro *[3,4] and *in vivo *[5-7]. While its ability to electrostatically bind and condense DNA [8], as well as facilitate endosomolysis to avoid lysosomal degradation [9,10], are major contributing factors to its relative greater efficacy over other non-viral vectors, very little is understood regarding the mechanism of internalization, the mode of transport throughout the cytoplasm, and the final entry into the nucleus.

Post endosomolysis, the mechanism of nuclear localization and subsequent entry of PEI polyplexes is not clear. Motor driven transport through the cytoplasm via interaction with microtubules has been suggested [11,12], however, the exact mechanism has not been elucidated. Active nuclear uptake is questionable [2] and various hypotheses of interactions with the nuclear pore complex, or the nuclear membrane itself, have been suggested [13]. Insufficient knowledge of these mechanisms limits the potential of PEI, and hence, further investigation is necessary to develop PEI towards becoming an effective therapeutic agent for gene therapy.

In the quest to bridge the efficiency gap between PEI and viral vectors, extensive research has been focused on the modification of PEI, with the aim of introducing novel properties to the vector. For a range of reviews discussing PEI modification, see [1,2,14]. Protein transduction domains (PTDs) are polypeptides that have the capacity to facilitate delivery and translocation of molecular cargo, both to and into the cytoplasm, and in some cases, the nucleus. The use of PTDs has significant potential for the basic investigation of cellular processes; moreover, they are of great interest because of their potential for the delivery of therapeutic molecules. While the exact mechanism of cellular internalization is unknown, PTDs have been suggested to mediate receptor-independent internalization via electrostatic interactions with negatively charged phospholipids and/or carbohydrate components on the cell surface [15]. Well-documented PTDs include the viral HIV-1 Tat DNA binding domain, and HSV-1 VP22 tegument protein, the Antennapedia DNA binding domain from *Drosophila*, and the synthetic polyarginine (Arg) peptides [16-19]. These PTDs have been used to deliver an extensive range of active molecules, including p53, Bcl-xL, *Cre *recombinase, and HOXB4 [16,20-25], to successfully influence a range of cellular processes.

In particular, synthetic Arg peptides have been demonstrated to be at least as effective as the HIV-1 Tat peptide [25-27]. Despite a well-documented ability to translocate membranes, the mechanism of Arg internalization and subsequent nuclear localization remains debatable. It has been traditionally accepted that Arg, and many other PTDs, demonstrate rapid cellular internalization (within minutes)[25], in addition to uninhibited uptake at 4°C [24]. This suggested an endocytic-, and receptor-independent internalization mechanism. Furthermore, Arg has been observed to localize within both cytoplasmic and nuclear compartments in fixed-cell studies [25-28]. Live-cell studies, however, show that Arg peptides exclusively localize within endocytic vesicles [29,30], and hence, the above cytoplasmic and nuclear observations have been suggested to be a function of fixation artefacts. While the use of the PTD Arg within a gene delivery strategy may be viable, further investigation is needed to extend the current understanding of Arg internalization within live cells. In addition, it is important to determine the fate of Arg peptides once internalized and, just as crucial, their potential for nuclear accumulation.

In this study, the ability of PEI and PEI-Arg bioconjugates to deliver plasmid DNA, in terms of cellular uptake, intracellular trafficking and biodistribution, and expression of exogenous DNA, was examined. More specifically, the efficiency of PEI and PEI-Arg polyplex-facilitated transfection was determined. The total percentage of cells with internalized reporter plasmid and the relative level of expression were examined using labelled DNA and a GFP reporter plasmid. In addition, the amount of plasmid internalized and expressed per individual cell was determined, in order to further characterize the efficiency of polyplex-facilitated DNA delivery. Intracellular trafficking pathways of both fluorescently-labelled DNA and polymers were studied, to investigate trafficking of PEI/pDNA and PEI-Arg/pDNA complexes and their ability to reach the nucleus, a pre-requisite for expression of the exogenous DNA. Finally, the effects of microtubules and mitosis were examined to determine the significance of their contribution to PEI and PEI-Arg facilitated gene expression. Our data suggest that the resulting expression of exogenous DNA by PEI bioconjugates is dependant on microtubule trafficking. Despite an increase in the amount of DNA internalized by PEI-Arg polyplexes, the lack of active transport mechanisms as a result of a different alternative internalization mechanisms, contributed to a very low PEI-Arg facilitated gene transfection and expression.

## Methods

### DNA constructs utilized in this study

The plasmid pCH110 was fluorescently labelled with the DNA intercalator YOYO-1 iodide for uptake analysis by PEI and PEI-bioconjugate polyplexes (see section below). The plasmid pEOTCGFP was used for quantitative expression analysis. It contains a mitochondrial ornithine transcarbamylase targeting signal with an in-frame GFP construct. Plasmids were purified from bacterial culture (DH5α) using Qiagen reagents and stored in deionised H_2_O at -20°C.

### Conjugation of poly-arginine peptides to PEI

Polyethylenimine (PEI; 25 kDa; Sigma) was conjugated to Arg peptides (RRRRRRRRRRRGC) using the heterobifunctional crosslinker N-succinimidyl 3-(2-pyridyldithio)propionate (SPDP; Sigma), using a protocol modified from [31]. In brief, 3 ml of a 500 mM PEI stock solution in HEPES buffer 1 (350 mM NaCl, 20 mM HEPES, pH 8) was added to 2 ml of 20 mM SPDP in dimethyl sulfoxide (DMSO), and incubated at room temperature on a orbital plate shaker overnight. Un-conjugated SPDP was removed by gel filtration using a G-25 Sephadex column equilibrated with HEPES buffer 2 (250 mM NaCl, 20 mM HEPES, pH 7.4), and was eluted in 3.5 ml of the same buffer. The degree of modification was determined by spectrophotometric analysis at 343 nm by release of pyridine-2-thione after reduction by excess dithiothreitol (DTT, 100 mM) for 30 min.

Peptide conjugation was completed by combining a 1 ml aliquot of PEI-SPDP solution with 4 mg of peptide at a 5-fold molar excess of peptide to PEI-SPDP. The peptide/PEI-SPDP solution was incubated at room temperature on an orbital plate shaker overnight. The extent of peptide conjugation to PEI-SPDP was determined by the release of pyridine-2-thione measured spectrophotometrically at 343 nm. PEI-peptide conjugates were stored at 4°C.

### Fluorescent labelling of DNA and PEI

Plasmid pCH110 was fluorescently labelled with the intercalating nucleic acid dye YOYO-1 iodide (diluted from 1 mM stock solution in DMSO; Molecular Probes). 500 μl of pCH110 (0.1 mg/ml) was combined with 100 μl of 10× TAE buffer and 400 μl of 10 μM YOYO-1 dye in TE buffer in a microcentrifuge tube. The solution was mixed for at least 1 h at room temperature in the dark, and stored wrapped in foil at -20°C.

PEI was fluorescently labelled with the amine reactive probe, Oregon Green 488 carboxylic acid succinimidyl ester *5-isomer* (Molecular Probes, [13]). PEI was diluted to a concentration of 10 mg/ml in 0.1 M sodium bicarbonate pH 8.3. A 1 ml aliquot of PEI solution was transferred into a microcentrifuge tube, and, 50 μl of the prepared probe (dissolved in DMSO to a final concentration of 10 mg/ml) was added. The solution was incubated at room temperature on an orbital plate shaker for 1 h protected from light, and subsequently stored at 4°C.

### COS-7 cell maintenance

The experiments described were performed *in vitro *using adherent African Green Monkey kidney fibroblast cells (COS-7). The COS-7 cell line was cultured in Dulbecco's Modified Eagle Medium (DMEM; Multicel Thermo Trace) supplemented with fetal bovine serum (to 5%; Thermo Trace) and penicillin/streptomycin (5000 U/ml each; CSL). Cells were grown at 37°C in a 5% CO_2 _atmosphere, and were passaged 3 times weekly for 4 weeks.

### Transfection using PEI

PEI and DNA solutions were prepared before each experiment at various molar ratios of PEI nitrogen (N) to DNA phosphate (P) (where 1 μg DNA equals 3 nmol of phosphate, and 1 μl of PEI stock contains 10 nmol of amine nitrogen, based on a 10 mM stock solution as defined in [5]). Based on this, the following calculation was used to determine the required volume of PEI from a stock solution [Volume of PEI of 10 mM stock (μl) = (desired N/P/3.3) × (μg DNA/1)].

For FACS analysis, cells were seeded in 24 well plates at 4 × 10^4 ^cells per well 24 h before transfection. 2 μg pDNA was initially diluted into 100 μl of 150 mM NaCl and vortexed, followed by the addition of polymer solution to reach a desired N/P ratio, as described above. The solution was vortexed and centrifuged briefly, and was allowed to complex at room temperature for 30 min, after which the transfection mixture was added to the cells.

For microscopic analysis, cells were seeded in a 6 well plate on top of a sterilized coverslip at 1–2 × 10^5 ^cells per well, 24 h before transfection. 200 μl of 150 mM NaCl was used to dilute 3 μg of pDNA. The transfection protocol for 24 well plates was then followed.

### Transfection using Lipofectamine 2000

2 × 10^5 ^cells were seeded in a 60 mm dish that contained a sterilised coverslip 24 h before transfection. Amounts indicated per dish are as follows: 4 μg of pDNA (pCH110; fluorescently labelled) and 10 μl of Lipofectamine 2000 (Invitrogen) was diluted in separate sterile microcentrifuge tubes, each containing 250 μl of incomplete DMEM (serum negative; penicillin/streptomycin negative; 3.67 g/l sodium hydrogen carbonate). Each of the solutions were gently inverted and incubated at room temperature for 5 min. The Lipofectamine solution was then added to the pDNA solution, mixed by inversion, and incubated for a further 20 min at room temperature. Lipofectamine/pDNA solution was added to cells, and after 4 h cells were washed three times with PBS, and media replaced with serum positive DMEM.

### Preparation for transfection with addition of nocodazole and aphidicolin

The microtubule depolymerizing agent nocodazole (10 mM stock in DMSO; Sigma) was used to investigate the role of microtubules on intracellular trafficking and GFP expression. Cells were seeded 24 h before transfection. 2 h before transfection, media was removed and replenished with fresh DMEM containing nocodazole (final concentration 10 μM, [32]). Cells were analysed by fluorescent microscopy 5 h post transfection. For analysis of GFP expression, cells were washed 3 times with PBS 4 h post transfection and replenished with fresh DMEM containing nocodazole (10 μM). Cells were analysed by FACS 24 h post transfection.

The cell cycle disrupting agent aphidicolin (10 mM stock in DMSO; Sigma) was used to determine the role of nuclear membrane breakdown on GFP expression [33]. Cells were seeded in DMEM containing 10 μM aphidicolin 24 h before transfection. 2 h before transfection, media was replaced with fresh DMEM containing 10 μM aphidicolin. 4 h post transfection, cells were washed and media replaced with fresh DMEM containing 10 μM aphidicolin. Cells were analysed by FACS 24 h post transfection.

### Preparation and visualization of live cell samples

The intracellular trafficking of polyplexes was studied by fluorescent microscopy. Cells were transfected with labelled DNA/PEI complexes and observed using the Olympus BX-50 fluorescence microscope fitted with a SPOT RT 3CCD camera (Diagnostic Instruments) and processed using SPOT Advanced software (version 3.4) at 1 h, 2 h, 3 h, 4 h, and 5 h post transfection.

Preparation of samples was as follows: Cells were seeded on top of sterilized coverslips and transfected as described above. 30 min prior to viewing cells, MitoTracker CMXRos (in DMSO; 10 mM, Molecular Probes) was added directly to cells in DMEM at a working concentration of 20 nM. Immediately before viewing, coverslips with cells were washed 4 times with PBS, and were lightly blot-dried by touching the coverslip on its edge to a tissue. The coverslip was gently placed, inverted, on a microscopic slide, and nail polish was used to seal the edges of the coverslip to the slide. Samples were viewed at 100× magnification by oil immersion.

### Preparation of fixed cell samples for immunofluorescence assay

An immunofluorescence assay was used to visualize microtubules. Cells were seeded on top of a sterilized coverslip at a cell density of 1 × 10^5 ^per well, 24 h prior to fixation. All subsequent steps were completed at room temperature. The media was removed, and the coverslip was washed 3 times with filtered PBS. (N/B – after each of the following steps, the coverslip was additionally washed 3 times with filtered PBS). Cells were fixed for 10 min with 1 ml of 4% paraformaldehyde. Cells were then permeabilized for 5 min with 1 ml of 0.2% Triton ×100 in PBS. 50 μl of a 1/100 dilution of mouse monoclonal anti-β-tubulin primary antibody (diluted in 0.2% Triton-3% BSA solution; Sigma) was added and allowed to incubate for 1 h. Finally, 50 μl of a 1/200 dilution of FITC-conjugated secondary antibody was added and incubated for a further 30 min. The coverslip was washed and mounted on a microscopic slide as described above.

### Fluorescence activated cell sorting (FACS) analysis

FACS analysis was used to quantify PEI and PEI-Arg facilitated cellular internalization and gene expression transfection efficiency. Cells were washed thoroughly 4 h post transfection with PBS to remove unbound and surface-bound polyplexes. After 24 h, each well was further washed twice with PBS, and trypsin was added to detach cells. Cells were resuspended and collected in PBS, and subsequently analysed using a FACS Calibur flow cytometer (Beckton Dickinson). Cytometric data was analysed using CELLQuest software. Cells were collected to a designated 10,000 events or 180 seconds of passage time.

## Results and discussion

### PEI-Arg bioconjugate effectively promotes pDNA uptake in a very high percentage of cells

Internalization of polyplexes was analysed in this study with the aim to determine both the proportion of cells within the population that display pDNA internalization, and the efficacy of internalization of labelled DNA per positively transfected cell. FACS analysis was used to examine internalization of PEI and PEI-Arg polyplexes, detected by the presence of fluorescently labelled pCH110, which had been complexed with polymer prior to transfection. In addition, the analysis investigated any potential trends that may occur across a range of N/P ratios from 0 through to 14, which encompasses a common range in which PEI is used throughout the literature.

**Figure 1:**
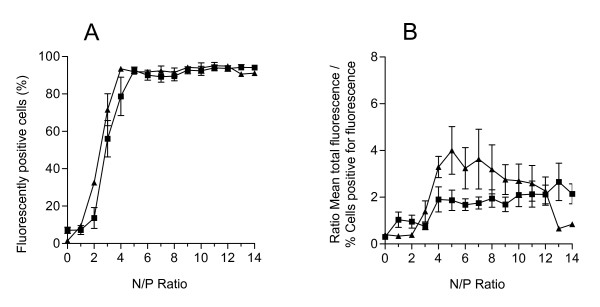
**Efficiency of PEI- and PEI-Arg polyplex internalization**. The percentage of fluorescently positive cells (A) and the relative amount of fluorescence per fluorescently positive cell (B) were calculated for polyplexes composed of PEI (■) and PEI-Arg (▲) complexed with YOYO1-labelled pCH110, as detected by FACS 24 h post transfection. Data points represented as mean values ± SEM (N = 3).

Both PEI and PEI-Arg polyplexes were internalized by 92.23% (± 4.66) and 92.75% (± 2.65) of cells respectively (Figure 1a) across the range of N/P ratios tested, but only after the N/P ratio was above 4. Below an N/P ratio of 4, internalization of both polyplexes is poor, and the percentage of cells fluorescing is almost negligible at N/P of 1. This correlates strongly with the neutralization in overall polyplex charge observed in gel mobility assays (data not shown), both supporting the reported data that a net positive polyplex charge is a prerequisite for efficient internalization [5].

Importantly, the addition of Arg peptides did not seem to negatively affect the uptake ability of native PEI. As both polyplex configurations displayed a high percentage of cells fluorescing (>92%), it is likely that uptake had reached a saturation point limited by the maximum possible exposure of polyplex to cells. Further enhanced polyplex uptake is likely to have been hampered by aggregation and layering of cells in culture.

### PEI-Arg polyplexes are internalized two-fold more efficiently than PEI polyplexes

The efficiency of polyplex internalization was determined by examining the relative amount of fluorescently labelled DNA internalized within each cell. This was calculated as a relative ratio by dividing the total mean fluorescence by the percentage of cells fluorescing. This ratio therefore gave an indication of mean fluorescence per individual cell, and hence provided a relative but sensitive means to detect changes in the actual amount of fluorescence per cell. Furthermore, a direct comparison of transfection by both polyplex types could be examined (Figure 1b). PEI polyplexes displayed a ratio of approximately 2.0 across the N/P ratios tested above N/P 3.0, however, PEI-Arg displays a doubling of ratio above PEI from N/P 4.0–8.0, which decreased above N/P 8. The number of PEI-Arg polyplexes internalized per cell was greater than that internalized by PEI polyplexes by approximately 2-fold. This therefore suggests that the addition of the Arg peptide enhances the amount of polyplexes internalized, and hence increases the amount of pDNA within the cell available to be delivered to the nucleus.

Interestingly, there was no significant difference in the percentage of cells positively fluorescing above an N/P ratio of 4 for PEI and PEI-Arg, and there was not a significant difference in the amount of polyplexes internalized across the N/P range tested (above N/P 4). Therefore, we surmise that the presence of a positive charge alone is necessary for internalization, and that the increased charge with greater N/P ratios does not further promote internalization. Furthermore, it is possible that the maximal polycationic compaction of pDNA has been reached, such that the complex size is reduced to a level that further positive charge it is no longer a factor in the translocation of DNA into the cell or into the nucleus. Both of these observations have important implications for the use of PEI and polyplexes *in vivo*. An increased net positive polyplex charge has a greater association with toxicity by extra- and intracellular non-specific charge-related interactions [34,35], and hence transfection with the lowest possible net polyplex charge that still facilitates efficient internalization is crucial to successful efficient delivery of exogenous DNA.

### PEI but not PEI-Arg polyplexes localize specifically to within proximity of the nucleus

Intracellular trafficking of PEI and PEI-Arg were analysed by fluorescence microscopy at hourly time intervals during the first five hours post transfection. An N/P of 8 was used for each transfection, a ratio previously established to be most suitable for microscopy analysis [13]. The intracellular trafficking of individually labelled PEI/pDNA and labelled pDNA/PEI polyplexes was initially analysed, from which the trafficking of labelled pDNA/PEI-Arg could be compared.

**Figure 2:**
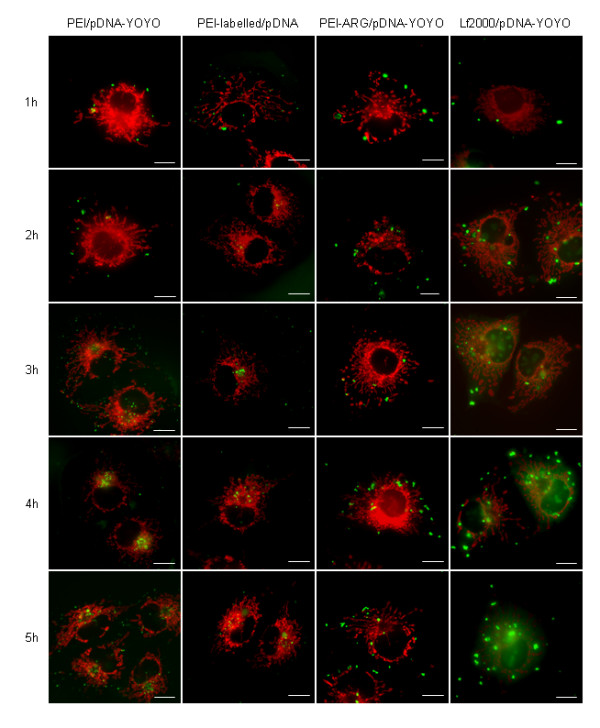
**Intracellular trafficking of PEI- and PEI-Arg polyplexes**. Time points were taken on the hour, for the first 5 hours post transfection, and viewed by fluorescent microscopy. Merged images were constructed from individual labelled-PEI-, or labelled-pDNA-, and MitoTracker fluorescent images, and are representative of a typical image obtained from five images taken per time point, from two independent experiments. Scale bar = 10 μm.

Within one hour, labelled pDNA/PEI complexes were seen to be interacting with the cell surface and within the cytoplasmic periphery (Figure 2 – PEI/pDNA-YOYO), with evidence of polyplex aggregation in specific areas of the cell surface, seen in Figure 2 (1 h). Complexes were seen to be internalized at 2 hours, and, within 4 hours, significant localization within the nuclear proximity was observed, highlighted by the accumulated fluorescence observed in the DNA and merged photographs (Figure 2 – PEI/pDNA-YOYO 4 h). The distribution of fluorescence was not uniform around the perinuclear region, but co-localized within a region corresponding to the microtubule organization center (MTOC), providing evidence of microtubule involvement in PEI trafficking. The MTOC is located adjacent to the nucleus, and is indirectly identified in the MitoTracker images (Figure 2) as a ring of densely stained mitochondria surrounding a region of markedly fewer mitochondria.

Although it was demonstrated that the labelled pDNA, which was presumed to be part of the PEI/pDNA-labelled polyplex, were localizing to the perinuclear/MTOC-associated region, it was essential to confirm that PEI itself, when complexed with fluorescently labelled DNA, was also trafficked to the MTOC-associated region. PEI was labelled with the fluorescent probe Oregon Green 488, complexed with unlabelled pDNA, and was viewed under the same time conditions (Figure 2 – PEI-labelled/pDNA). PEI-labelled/pDNA polyplexes were seen to be within the cytoplasm at the first hour, which was observed earlier than PEI/pDNA-YOYO polyplexes. Interestingly, MTOC-associated accumulations were also seen as early as two hours, which was detected earlier than PEI/pDNA-YOYO polyplexes. We suggest that this observation is due to uncomplexed PEI being rapidly internalized prior to PEI/pDNA polyplexes, providing visual evidence in support of the work of Boeckle et al. [35], who demonstrated the importance of un-complexed PEI in promoting efficient transfection with PEI.

The commercially available cationic lipid Lipofectamine 2000 was used to determine if MTOC-associated localization of PEI/pDNA was a property of PEI polyplexes alone (Figure 2 – Lf2000/pDNA-YOYO). In contrast to cationic polymer-mediated delivery, lipoplexes did not display similar MTOC-associated accumulation, but appeared to be randomly dispersed throughout the cell. Interestingly, lipoplexes did show some nuclear localization as early as 3 h post transfection, whereas PEI polyplexes did not display any significant nuclear localization over the entire five hours analysed. Lack of specific MTOC-associated accumulation by lipid mediated transfection suggests that localization exhibited by PEI polyplexes to the MTOC-associated region might indeed be PEI specific.

The Arg peptide was conjugated to PEI to enhance the membrane transductional properties of PEI. Analysis of PEI-Arg complexed with labelled pDNA revealed that while polyplexes were observed inside the cell periphery (within the same time period as PEI throughout the time course studied), no MTOC-associated fluorescence accumulation was observed (Figure 2 – PEI-Arg/pDNA-YOYO). Even at the 5 h time point, labelled pDNA was found to be widely distributed throughout the cytoplasm. We therefore conclude that differences in the internalization mechanisms between both PEI and PEI-Arg ultimately affect the subsequent intracellular trafficking of the respective polyplexes.

It is accepted that a significant feature of PEI which contributes to its gene delivery efficiency is its ability to act as a proton sponge within the endosome [9,10,36]. Endosomes become acidic as they migrate and mature into late endosomes, which in turn initiates proton capture by PEI, facilitating the proton sponge effect, and subsequently initiating endosomolysis. The absence of MTOC-associated localization by PEI-Arg/pDNA polyplexes, and hence lack of PEI-Arg/pDNA trafficking to the nuclear periphery, suggests that the Arg mediated internalization mechanism is different to that of PEI polyplexes alone. We suggest that the Arg internalization does not follow a traditional endocytotic and endosomal recycling pathway, but is internalized by an alternative mechanism, such as macropinocytosis, or in particular a lipid raft/caveolar-like vesicular uptake, whereby the vesicles are slowly internalized [37], and do not become acidified [38,39], hence avoiding lysosomal fusion and subsequent degradation of the contents within. This mechanism is likely to explain the random distribution of Arg polyplexes, which was morphologically not dissimilar to the nocodazole-treated cells transfected with PEI/pDNA. As opposed to PEI polyplexes, PEI-Arg polyplexes may be entrapped within such vesicles, due to its likely inability to promote vesicle lysis. Caveolae-mediated internalization is attractive as an alternative for DNA delivery, as a significant proportion of delivered DNA is degraded within the lysosome post-endosomal transport before it becomes available to the nucleus. Further analysis will be necessary to ascertain the true characteristics of these vesicles.

### Intact microtubules are essential for specific perinuclear/MTOC-associated localization of PEI/pDNA polyplexes

**Figure 3:**
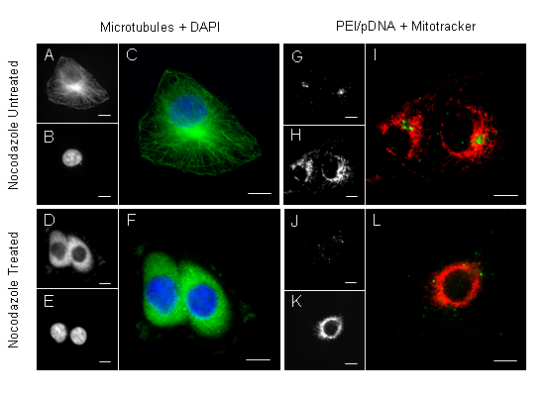
**Effect of microtubule disrupting agent, nocodazole, on microtubule morphology and PEI polyplex trafficking**. Untreated (A) and nocodazole treated cells (D) displaying normal and disrupted microtubules, highlighted by anti-β-tubulin visualized using FITC secondary antibody. Nucleus stained with DAPI (B, E) and merged immunofluorescence displayed as (C, F). Distribution of PEI/pDNA-YOYO in untreated (G) and nocodazole treated (J) cells with MitoTracker staining (H, K). Merge MitoTracker and PEI/pDNA-YOYO in untreated (I) and nocodazole treated (L) cells. Scale bar = 10 μm. Images represent typical result of two individual slides viewed for each sample, analyzed 5 h post transfection.

The microtubule depolymerizing agent nocodazole was utilized to investigate potential association between microtubules and active PEI intracellular trafficking. Microtubules within nocodazole-treated cells were visualized by immunofluorescence using fluorescent microscopy to examine the impact of disrupted microtubules on PEI trafficking (Figure 3). Untreated mitochondrial, PEI/pCH110-YOYO, and merged images depict the accumulation of PEI at the MTOC-associated region. The microtubule controls, showing immunofluorescence of microtubules, nucleus, and merged images highlight a filamentous-microtubule structure throughout the cell and an intense staining of microtubule accumulation within a defined MTOC-associated perinuclear region. Nocodazole-treated cells containing PEI/pDNA-YOYO polyplexes display a random distribution of labelled complexes throughout the whole cell, as compared to controls that display MTOC-associated fluorescence accumulation. Furthermore, the filamentous morphology of mitochondria stained with MitoTracker was seen to be disrupted, becoming evenly distributed around the nucleus. The nocodazole-treated cells displayed disrupted microtubules, highlighted by the loss of the filamentous structure and replaced by a uniform fluorescence throughout the cell. Randomly dispersed fluorescence of PEI polyplex distribution was observed in nocodazole-treated cells (Figure 3E), as compared to the predictable PEI accumulation at the MTOC-associated region in non-treated cells (Figure 3B). The significant differences in morphology of microtubules and PEI accumulations observed between the treated and untreated cells, strongly indicated an albeit indirect correlation between the disruption of microtubules and loss of PEI trafficking.

As PEI trafficking to the MTOC-associated region occurred within 4 hours, and that PEI is thought to begin endosomal escape at approximately this time [13], we suggest that PEI is encapsulated within endosomal vesicles at least until this time point. The association of endosomal trafficking and microtubules is relatively well established, especially in connection with receptor-mediated endocytosis and subsequent receptor-ligand sorting [40]. We therefore hypothesize that the active trafficking of PEI polyplexes is in fact not due to a direct association between PEI and microtubules, but an indirect association, facilitated by PEI localization within the endosomal compartment at the time of endosomal transport.

### GFP expression is largely dependent on proximal nuclear accumulation of PEI, and is greatly enhanced by mitotic events

Quantitative analysis of gene expression was undertaken to determine the efficiency of transfection for each polyplex bioconjugate. Positive GFP expression was detected by FACS analysis 24 hours post transfection. Transfection efficiency was measured by two analyses; (1) percentage of cells that expressed GFP in the population, and, (2) the amount of fluorescence per GFP positive cell. The latter was used to correlate the intensity of GFP fluorescence with the relative number of plasmid expressing GFP. Both measures of efficiency are useful and important as it depends on the therapeutic strategy employed to treat a particular disorder. Under certain circumstances, it would be important to obtain high gene expression per cell, while other strategies benefit from the maximum percentage of cells transfected.

**Figure 4:**
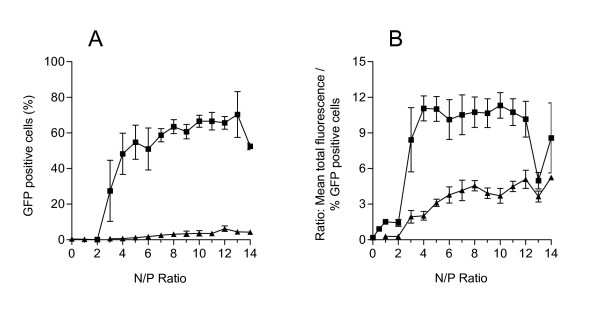
**Gene expression efficiency mediated by PEI- and PEI-Arg polyplexes**. The percentage of GFP positive cells (A) and the relative amount of fluorescence per GFP positive cell (B) was calculated for polyplexes composed of PEI (■) and PEI-Arg (▲) complexed with pEOTCGFP, as detected by FACS 24 h post transfection. Data represented as mean values ± SEM (N = 3).

As it was initially hypothesized that the Arg peptide would facilitate and enhance translocation across the plasma membrane, there would be an increased amount of PEI-Arg polyplexes available for nuclear uptake. PEI polyplexes facilitated significantly greater GFP expression, with a significantly higher proportion of cells expressing GFP than PEI-Arg (Figure 4A). GFP expression for each polyplex type was evident only after an N/P ratio of 3, and was maintained until N/P 14. In a comparison of the extent of GFP expression efficiency per cell (Figure 4B), PEI displayed between 3- and 5-fold more GFP fluorescence per transfected cell than PEI-Arg. Surprisingly, despite facilitating more DNA internalized per cell, PEI-Arg mediated expression was very inefficient, displaying no more than 5% of GFP positive cells across the entire N/P range examined.

**Figure 5:**
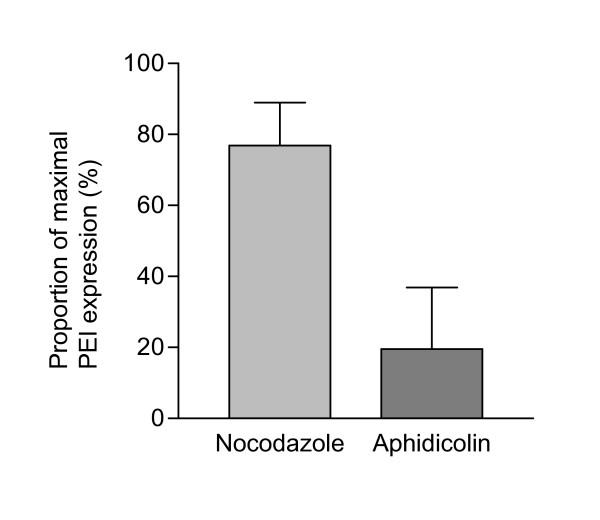
**Effect of nocodazole and aphidicolin on PEI polyplex mediated GFP expression**. Expression of GFP 24 h post transfection in the presence of nocodazole (light bar), or aphidicolin (dark bar), as a percentage of optimal PEI/pDNA mediated GFP expression at N/P 8. Data obtained as detected by FACS 24 h post transfection, and represented as mean values ± SEM (N = 2).

The relative high PEI-/low PEI-Arg-facilitated expression indicates a correlation with the presence, and absence, of MTOC-associated accumulation respectively, and hence a possible correlation between microtubule-dependent intracellular trafficking of polyplexes and GFP expression. GFP expression analysis of nocodazole treated cells displayed a reduction of more than 20% of maximal PEI expression (Figure 5). This suggests that GFP expression is not exclusively facilitated by microtubule-dependent trafficking. Taking this into consideration, we expected that the transfection efficiency of PEI-Arg would have been greater. As PEI-Arg-facilitated GFP expression averaged 6-fold less GFP-positive cells (Figure 4B), there must be further limitations affecting PEI-Arg that were not directly evident in this study. This however may be further evidence for the retention of PEI-Arg polyplexes within non-endocytic vesicles. We hypothesize that expression of DNA complexed with PEI-Arg entrapped in these vesicles is hampered, even if they had been engulfed within the nucleus during mitosis.

The occurrence of significantly higher gene expression obtained in rapidly dividing cells compared with slow dividing, or post mitotic cells [1,2] denotes the importance of the breakdown of the nuclear membrane during mitosis. To investigate the effect of inhibiting nuclear membrane breakdown on GFP expression, cell cycle was arrested at the G1/S checkpoint by the addition of the anti-mitotic agent aphidicolin. Aphidicolin restricts cells in the S phase by inhibiting DNA polymerase α, and hence allows cells at various stages of the cell cycle to accumulate at the G1/S border [41]. GFP expression facilitated by PEI in the presence of aphidicolin was inhibited up to 80% of maximal PEI-facilitated GFP expression of control cells (Figure 5), which was comparable to results obtained from an aphidicolin assessment on cation-lipid mediate gene delivery [33]. The level of inhibition observed indicates that the efficiency of gene delivery is significantly but not entirely dependent on mitotic events, as gene expression was not completely eliminated (8.19% ± 3.95 [SEM, N = 2]; percentage of GFP positive cells treated with aphidicolin). It would be of interest to inhibit microtubule transport in post-mitotic cells to further define the role of microtubules in PEI trafficking.

In light of this, the fact that some cells that do not undergo cell division, or those that have been prevented from dividing as seen in this study, can be transfected, indicates there must be at least one mechanism of polyplex nuclear entry in the presence of an intact nuclear envelope. Godbey *et al. *[13] hypothesize that polyplex interactions with phospholipids may facilitate nuclear uptake. The coating of cationic polyplexes with anionic lipids may facilitate interactions with the lipid and nuclear membrane, which may ultimately release the polyplex into the nucleus. They speculated that the coating could be either via interactions with free phospholipids that are constantly being synthesized for membrane regeneration, or that tight interactions with endosomal phospholipids after endosomolysis may be responsible. Another potential route is via the nuclear pore complex (NPC), which may facilitate diffusive or active uptake of polyplexes [1]. The latter, however, seems to be unlikely, as only particles smaller than 9 nm in diameter can readily diffuse through the NPC, which excludes the significantly larger polyplexes. Furthermore, active transport through the NPC requires the activation of energy-dependent transport mechanisms, a step in which is unlikely to occur for PEI-mediated transfection.

## Conclusion

Internalization of polyplexes was observed to be highly efficient for both PEI and PEI-Arg. The conjugation of Arg to PEI did not negatively affect internalization, and we have demonstrated that the attachment of Arg peptides to PEI increased polyplexes internalized, resulting in more DNA per cell. We speculate however that the attachment of the Arg peptides alters the mechanism by which PEI polyplexes enter the cell, and that this alternative mechanism is responsible for the altered biodistribution of PEI-Arg/pDNA polyplexes.

Active motor driven transport of PEI by microtubules has been suggested previously [11]. Conversely, we suggest that PEI trafficking is solely a consequence of PEI-encapsulated endosomal transport facilitated by microtubules, rather than a direct PEI-microtubule association. Therefore, the differences observed between the efficacy of PEI and PEI-Arg is likely to be largely due to the absence of microtubule trafficking of PEI-Arg polyplexes.

From this, we surmise that the absence of microtubule-facilitated trafficking of PEI-Arg polyplexes is the result of a lack of PEI-like endosomal compartmentalization, ultimately because of a difference in internalization mechanism. Moreover, this alternative internalization ultimately results in the significant reduction in gene expression observed, compared to that seen with PEI/pDNA polyplexes, and is likely due to a retention or entrapment within such vesicles. Such entrapment further supports the observation of cytoplasmic vesicular retention of Arg peptides in live cell studies [29,30].

Finally, we hypothesize that microtubules do not directly influence nuclear uptake, but facilitate proximal nuclear accumulation so that there is a greater probability of either nuclear capture during mitosis, or of direct interaction of polyplexes with the nuclear membrane itself.

Conjugation of peptides to PEI offers a promising approach to improve the transfection efficiency and ultimately the viability of an effective non-viral gene therapy strategy, despite some of the obstacles identified in this study. It can be speculated that clinical gene therapy applications will involve the use of a multi-component vector; each constituent contributing a specialized property that utilizes an individual innate cellular pathway or function, to provide specific and efficient delivery of therapeutic genes to the nucleus.

Continued investigation is needed to further characterize cellular pathways of PEI and other non-viral vectors. This will in turn, further identify important bottlenecks of transfection efficiency allowing new and improved strategies to be developed. Only with the support of gene delivery research will the gap between the promise and the clinical application of gene therapy be realized for many currently untreatable genetic diseases.

## Competing interests

The author(s) declare that they have no competing interests.

## Authors' contributions

SRD completed all the technical molecular work, and drafted the manuscript. CKC conceived of the study, participated in its design and coordination, and helped to draft the manuscript. Both authors read and approved the final manuscript.
